# La fibrillation atriale dans trois centres cardiologiques de référence de Dakar: données sénégalaises de l’enquête du registre AFRICA

**DOI:** 10.11604/pamj.2022.43.112.31397

**Published:** 2022-10-31

**Authors:** Khadimu Rassoul Diop, Cheikh Ahmadou Bamba Samb, Adama Kane, Joseph Salvador Mingou, Serigne Mor Beye, Youssou Diouf, Simon Antoine Sarr, Fatou Aw, Papa Guirane Ndiaye, Cheikh Ahmadou Bamba Mbacke Diop, Malick Bodian, Mouhamadou Bamba Ndiaye, Maboury Diao, Anicet Kassi Adoubi, Abdoul Kane

**Affiliations:** 1 Service de Cardiologie, Hôpital Aristide Le Dantec, Dakar, Sénégal,; 2 Service de Cardiologie, Centre Hospitalier Régional de Saint-Louis, Saint-Louis, Sénégal,; 3 Service de Cardiologie, CHU de Bouaké, Bouaké, Côte d´Ivoire,; 4 Service de Cardiologie, Hôpital Dallal Jamm, Dakar, Sénégal

**Keywords:** fibrillation atriale, AFRICA, insuffisance cardiaque, valvulopathie, Sénégal, Atrial fibrillation, AFRICA, heart failure, valvulopathy, Senegal

## Abstract

**Introduction:**

la fibrillation atriale (FA) est le trouble du rythme cardiaque le plus fréquent. Sa prévalence est sous-estimée en Afrique, d´où l´initiation du registre AFRICA (Atrial Fibrillation Registre In Countries of Africa). Notre étude avait pour objectif de décrire, dans le cadre du registre AFRICA, les aspects épidémiologiques, cliniques, paracliniques, thérapeutiques et évolutifs de la fibrillation atriale en Afrique particulièrement au Sénégal.

**Méthodes:**

il s´agissait d´une étude transversale, rétrospective multicentrique, menée du 1^er^ janvier au 31 décembre 2017, dans trois services de cardiologie de référence du Sénégal.

**Résultats:**

cent soixante-huit patients, avec un âge moyen de 63 ans, ont été sélectionnés; soit une prévalence hospitalière de 5,99%. On notait une prédominance féminine (sex-ratio 0,69). L´hypertension artérielle (HTA) était le facteur de risque le plus fréquent (24,4%). L´insuffisance cardiaque était la circonstance de découverte la plus fréquente (59,52%). La FA était persistante dans 52,24%, la FA valvulaire représentait 31%, et était plus fréquente chez les jeunes (p= 0,005). La fonction systolique du ventricule gauche était altérée dans 55,7%, l´oreillette gauche était dilatée dans 70,83%. La stratégie visant à réduire la fréquence cardiaque était la plus utilisée. Les patients ayant un CHA2DS2VASC ≥ 2 ont bénéficié d´une anticoagulation faite d´HBPM avec en relais oral (le plus souvent par de l’Anti Vitamine K (AVK)). Les complications étaient dominées par l´insuffisance cardiaque (66,6%) et AVCI (28%).

**Conclusion:**

la fibrillation atriale (FA) est le trouble du rythme cardiaque le plus fréquent. Elle pose un réel problème de santé publique.

## Abstract (en)

**Introduction:**

atrial fibrillation (AF) is the most common cardiac rhythm disorder. Its prevalence is underestimated in Africa, hence the initiation of the Atrial Fibrillation Registry In Countries of Africa (AFRICA). The aim of our study was to describe, within the framework of the AFRICA registry, the epidemiological, clinical, paraclinical, therapeutic and evolutionary aspects of atrial fibrillation (AF) in Africa, particularly in Senegal.

**Methods:**

we performed a cross-sectional, retrospective, multicentric study conducted from January 1^st^ to December 31^st^ 2017, in three referral cardiology wards in Senegal.

**Results:**

one hundred and sixty-eight patients, with a mean age of 63 years, were selected, representing a hospital prevalence of 5.99%. There was a predominance of women with sex-ratio of 0.69. High blood pressure was the most frequent risk factor (24.4%). Heart failure was the most frequent circumstance of discovery (59.52%). AF was persistent in 52.24% and valvular AF accounted for 31% and was more frequent in young people (p= 0.005). Left ventricular systolic function was impaired in 55.7%, the left atrium was dilated in 70.83%. The strategy to reduce heart rate was the most used. Patients with CHA2DS2VASC ≥ 2 received anticoagulation with LMWH and oral relay maid mostly of VKA. The complications were dominated by heart failure (66.6%) and ischemic stroke cerebral (28%).

**Conclusion:**

atrial fibrillation (AF) is the most frequent cardiac rhythm disorder. It is a major public health concern.

## Introduction

La fibrillation atriale est caractérisée par une activation atriale désorganisée avec comme conséquence une détérioration de la fonction mécanique des oreillettes et une stase sanguine avec risque accru de thrombose in situ et d´embolie [1]. Du point de vue épidémiologique, la fibrillation atriale est la plus fréquente des arythmies avec une prévalence estimée à 0,1 à 4% selon les populations et selon les pays [2,3].

Cette prévalence augmente avec l´âge. Ainsi, plus d´un quart des individus de plus de 40 ans sont à risque de développer cette arythmie [4,5]. Si cette affection suscite autant d´intérêt c´est parce qu´elle peut se compliquer d´Accident Vasculaire Cérébrale (AVC) et d´insuffisance cardiaque. Cependant, peu de données existent en Afrique, d´où l´initiation du registre AFRICA (Atrial Fibrillation Registre In Countries of Africa), dont les objectifs étaient de décrire les aspects épidémiologiques, cliniques, paracliniques, thérapeutiques et évolutifs de la FA en Afrique particulièrement au Sénégal.

## Méthodes

**Cadre de l´étude:** il s´agissait d´une étude multicentrique, transversale, analytique, descriptive et rétrospective réalisée dans trois services de cardiologie de référence du Sénégal (Hôpital Aristide Le Dantec, Hôpital Général Idrissa Pouye et Hôpital Principal de Dakar).

### Population d´étude

***Critères d´inclusion*** ont été inclus dans cette étude tous les dossiers de malades hospitalisés pour fibrillation auriculaire, sans aucune distinction de sexe, de race ni d´âge, ou ayant développé en cours de l´hospitalisation une FA documentée à l´électrocardiogramme (ECG) durant la période de l´étude.

***Critères de non inclusion*** n´ont pas été inclus dans ce travail tous les dossiers de patients porteurs de FA non hospitalisés dans ces services durant la période de l´étude.

### Méthode d´étude

**Type et période d´étude:** une étude multicentrique, transversale, analytique, descriptive et rétrospective allant du 1^er^ janvier 2017 au 31 décembre 2017 avait été menée.

**Analyses statistiques:** les données avaient été saisies avec le logiciel EXCEL version 2010. L´analyse des données a été effectuée avec le logiciel SPSS version 23. L´étude descriptive était réalisée avec le calcul des fréquences et proportions pour les variables qualitatives et le calcul des moyennes pour les variables quantitatives. L´étude analytique était faite avec les tableaux croisés. Pour comparer les fréquences, nous avions utilisé le test du Khi-deux de Pearson ou le test exact bilatéral de Fisher selon leurs conditions d´applicabilité avec un seuil de significativité p = 0,05.

## Résultats

### Aspects épidémiologiques

Cent soixante-huit patients (168) ont été sélectionnés soit une prévalence hospitalière de 5,99%. L´âge moyen était de 63± 15,48 ans; 87,5% de nos patients avaient plus de 45 ans et les tranches d´âge les plus représentées étaient celles de 67 à 76 ans dans 61%. On notait une prédominance féminine avec un sex-ratio de 0,69. Dans notre population d´étude, 47,92% étaient illettrés et 25% parmi les instruits (52,08%) avaient le niveau primaire. L´HTA était le facteur de risque le plus fréquent, rencontrée dans 24,4%.

### Aspects cliniques

L´insuffisance cardiaque était la circonstance de découverte la plus fréquente soit 59,52%, suivie des palpitations dans 30,95%. Le mode de survenue était brutal dans la majorité des cas (38,68%) et le stress constituait le facteur déclenchant majoritaire (38%). Dans notre série on retrouvait 2,97% cas d´anémie clinique, 2,38% cas de cancer et 1,78% de cas d´hyperthyroïdie. On retrouvait 40,6% de patients ayant un score EHRA 3 et 30,3% un score EHRA 4.

### Aspects paracliniques

L´anémie était l´anomalie biologique la plus fréquente (14,8%), suivie de l´insuffisance rénale dans 7,73%. On retrouvait une FA dans 92,26% à l´ECG, avec une moyenne de la fréquence ventriculaire à 109 ± 33,98 cycles/min pour des extrêmes allant de 45 à 250 cycles /min. On notait une prédominance des FA à petites mailles (92,2%), la FA persistante prédominait et représentait 52,24% des cas suivie de la FA permanente dans 25% des cas. Parmi les troubles de la conduction, un bloc auriculo ventriculaire était retrouvé dans 3,57% et un h émi bloc antérieur dans 2,38%. Comme autres troubles du rythme, on retrouvait une Extra Systole Ventriculaire dans 4,8% et un (01) cas de Tachycardie ventriculaire non soutenue. La FA non valvulaire était retrouvée dans 69,05%, mais la FA valvulaire était plus fréquente chez les sujets de moins 45 ans (p de Pearson =0,0001) (Tableau 1), avec une nette prédominance de l´atteinte mitrale dans 30,76%. On retrouvait à l´échocardiographie Doppler transthoracique une fraction d´éjection du ventricule gauche altérée dans 55,7%, une dilatation de l´oreillette gauche dans 70,83%, une cardiopathie sous-jacente dans 85,71%; dominée par les cardiopathies valvulaires dans 31% et hypertensives dans 24,4%; un thrombus intracavitaire était retrouvé dans 5,95% et une HTAP dans 23,8%.

**Tableau 1:** répartition des fibrillations atriales valvulaires ou non selon l´âge

Classes d´âge	FA valvulaires		Total
	**Non**	**Oui**	
Moins de 45 ans	01	18	19
Plus de 45 ans	115	34	149
Total	116	52	168

### Aspects thérapeutiques

L´objectif thérapeutique était de ralentir la fréquence cardiaque chez 65% des patients, avec une utilisation prédominante de la Digoxine (42,85%) suivie des Beta bloquants (22,61%). Dans 21% il était de resinusaliser le rythme et dans 14% aucun objectif n´avait été fixé. Cinq (05) cas soit 2,97%, de restauration du rythme ont été notés, par de l´Amiodarone. Cent trente-six (136) soit 81% patients avaient un score de CHA2DS2VASC ≥ 2. Tous les patients, à FA valvulaires et les non valvulaires ayant un CHA2DS2VASC supérieur ou égal à 2 ont bénéficié d´une anticoagulation faite d´HBPM avec en relais oral fait d´Anti Vitamine K dans 75%. L´anticoagulant oral direct (AOD) n´a été utilisé que chez un (01) seul patient. Les motifs du choix d´anticoagulant étaient non précisés dans 76,19%, chez un (01) patient soit 0,59%, le motif était une préférence des AOD.

Des complications étaient retrouvées chez 72,17% des patients. Elles étaient d´ordre hémodynamique (insuffisance cardiaque dans 66,6%) et thromboembolique, notamment un Accident Vasculaire Cérébral Ischémique dans 28%. Deux cas de décès et deux cas d´accident hémorragique avaient, également, été notés dans notre série.

## Discussion

Les limites de notre étude résident dans son caractère rétrospectif.

Dans notre étude nous avons retrouvé une prévalence hospitalière de 5,99%, proches des données retrouvées aux États-Unis ou dans l'Union européenne, où la prévalence est entre 5-6% [6-9]. Néanmoins on devrait noter un sous diagnostic de la FA, car seules les formes symptomatiques sont hospitalisées en milieu cardiologique minorant ainsi la prévalence exacte de la FA. Bien que ne reflétant pas la prévalence exacte de la FA dans la population générale, ces données hospitalières donnent au moins un aperçu sur la réalité de ce trouble du rythme cardiaque.

Dans notre étude seuls 12,5% étaient âgés de moins de 45ans, ce qui ne concorde pas avec les données retrouvées en Afrique subsaharienne où les patients atteints de FA ont tendance à être plus jeunes [10]. Ceci pourrait être en rapport avec la transition épidémiologique qui prévaut en Afrique subsaharienne, les facteurs de risque et les complications de la FA qui en découlent ont changé et sont en augmentation. La prévalence de la FA augmente avec l´âge [10].

Dans notre série, l´insuffisance cardiaque était la circonstance de découverte la plus retrouvée (59,52%) suivie des palpitations dans 30,95%. Dans l´étude Framingham on retrouvait comme signes cliniques de la FA des palpitations, de la fatigue, des vertiges et une dyspnée à l'effort en rapport avec une insuffisance cardiaque [9]. Cette discordance peut être expliquée, d´une part, par le caractère subjectif des signes cliniques mais d´autre part par le polymorphisme clinique de la FA.

Notre enquête montrait que 14,8% de nos patients avaient une anémie; 7,73% une insuffisance rénale et 1,78% une hyperthyroïdie. Ceci peut être expliqué par une prédominance des sujets âgés dans notre population qui ont tendance à avoir diverses comorbidités. Cependant la prévalence de l´hyperthyroïdie, concordante avec les données de l´étude Framingham (1,5%) [9], semble être sous-estimée dans notre étude, car seule 7,14% des patients avaient bénéficié d´un dosage des hormones thyroïdiennes, expliqué probablement par la non systématisation de cette exploration dans la FA.

La prédominance de la FA persistante et permanente dans notre étude est retrouvée dans d´autres pays d´Afrique subsaharienne comme au Cameroun [11]. Certaines études suggèrent le contraire aux États-Unis et en Europe où la forme permanente de FA pourrait être moins courante (prévalence estimée de 30% à 40%). Ceci peut être expliqué en partie par la disparition des cardiopathies valvulaires dans ces pays développés [12-14]. D´autre part, nos patients peuvent progresser plus rapidement vers une FA persistante puis permanente en raison de la probabilité réduite de détection, lorsque la FA est paroxystique, il y a un recours moins fréquent aux thérapies de contrôle du rythme pour restaurer et maintenir le rythme sinusal, mais aussi la fréquence élevée des cardiopathies valvulaires dans nos régions[10].

Cinquante-deux (52) patients soit 31% présentaient une FA valvulaire (avec une prédominance de l´atteinte mitrale dans 30%) et 69% une FA non valvulaire. Des données similaires sont retrouvées à Bamako, Yaoundé [11,15]. Ces résultats ne sont pas conformes aux réalités des pays en voie de développement où la valvulopathie rhumatismale constitue une cause prépondérante de la fibrillation auriculaire [16]. Néanmoins ils montrent que la valvulopathie rhumatismale, certes en baisse, reste encore d´actualité car demeure une cause non négligeable de FA surtout en Afrique. La prédominance de FA non valvulaire, par contre, pourrait être expliquée par la prédominance des sujets âgés dans notre population d´étude, chez qui la FA non valvulaire prédomine.

Notre enquête a révélé que la possibilité de développer une FA valvulaire était fonction de l´âge car les sujets de moins de 45 ans avaient une prévalence plus élevée de FA valvulaire que les sujets de plus de 45 ans, (avec un p= 0,0001). En raison de la prévalence plus élevée des cardiopathies rhumatismales, la FA pourrait toucher les personnes en Afrique à un plus jeune âge [10,16]. Ceci serait en rapport avec la forte incidence des infections à streptocoques sous-traitées en Afrique contribuant ainsi à la charge élevée de fibrillation auriculaire valvulaire associée aux cardiopathies rhumatismales [10,16].

L´objectif de la prise en charge rythmologique était de ralentir la fréquence cardiaque chez 64% de ces patients et de resinusaliser chez 21%. Des données similaires ont été rapportées à Yaoundé où le contrôle de la fréquence cardiaque était la stratégie la plus adoptée (83,7%) [11]. A Nairobi, le contrôle de la fréquence cardiaque était la stratégie privilégiée chez 78% des patients [17]. Le contrôle de la fréquence cardiaque semble donc être la stratégie de traitement la plus adoptée, comparée au contrôle du rythme pour la prise en charge de la FA chez la plupart des cliniciens traitant des patients atteints de FA en Afrique[10]. Cette approche de traitement contraste avec la gestion de la FA dans les pays développés, où les médecins adoptent généralement le contraire, le contrôle du rythme prédomine [18]. Ceci peut être expliqué par une prévalence élevée de la FA permanente en Afrique lié à un retard diagnostic mais aussi au fait que presque 71% des patients avaient une dilatation de l’oeil gauche (l´OG) à l´échocardiographie transthoracique (l´ETT).

Un (01) seul patient avait bénéficié d´une cardioversion électrique, cette utilisation réduite de la cardioversion électrique dans notre étude serait liée au fait qu´il n´avait qu´un seul patient qui présentait une instabilité hémodynamique. Ce qui reste conforme aux nouvelles recommandations sur la FA [19]: la cardioversion électrique est recommandée chez les patients en instabilité hémodynamique aigue pour restaurer la fonction cardiaque, recommandation (IB).

Le rythme sinusal était restauré chez cinq (05) patients soit 2,97%. Ce faible pourcentage de restauration du rythme sinusal peut être expliqué par le fait que, le contrôle du rythme était la stratégie de traitement la moins adoptée dans la prise en charge des sujets atteints de FA dans notre étude. L´utilisation prédominante des AVK au détriment des AOD a été rapportée dans notre étude comme à Bamako [15]. La tendance prédominante, quant à elle, d´utilisation des AVK au détriment des AOD, alors que les avantages potentiels des AOD par rapport aux AVK pourraient être; qu´ils n´exigent pas de contrôle de routine de l´International Normalized Ratio (INR), ou qu´ils n´aient pas d´interactions avec les aliments ou les médicaments peuvent être prescrits à doses fixes, serait liée à l´inaccessibilité (relative) des AOD. Les inconvénients des AOD par rapport aux AVK comprennent leur coût, les rendant inabordables pour de nombreux patients et l'absence actuelle d'un antidote spécifique [20]. De plus, ils ne sont pas indiqués pour la prévention des accidents vasculaires cérébraux chez les patients présentant une FA valvulaire [21]. En outre les AVK génériques étant disponibles à moindre coût, ils devraient, alors, rester la norme dans la région.

Des complications étaient notées chez 72,17% de la population. Elles étaient dominées par les complications hémodynamiques (66,66%) suivies des complications thrombo-emboliques (29,41%) dont 28,43% d´AVCI. La FA est associée, à long terme, aux risques de complications notamment cardiaques et cérébrales comme le montre l´étude Framingham [3]. Dans notre étude, la découverte parfois tardive, et le retard diagnostique de la FA pourraient expliquer cette prévalence élevée des complications.

## Conclusion

La fibrillation atriale (FA) reste une réalité épidémiologique dans nos régions. Sous diagnostiquée, elle continue de constituer un réel problème de santé publique avec des complications importantes. Sa prise en charge est marquée par une utilisation prédominante des AVK au détriment des AOD liée à l´inaccessibilité (relative) de ces derniers.

## References

[1] Aimé-Sempé C, Folliguet T, Rücker-Martin C, Krajewska M, Krajewska S, Heimburger M (1999). Myocardial cell death in fibrillating and dilated human right atria. J Am Coll Cardiol.

[2] Benjamin EJ, Levy D, Vaziri SM, D´Agostino RB, Belanger AJ, Wolf PA (1994). Independent risk factors for atrial fibrillation in a population-based cohort. The Framingham Heart Study. JAMA.

[3] Benjamin EJ, Wolf PA, D´Agostino RB, Silbershatz H, Kannel WB, Levy D (1998). Impact of atrial fibrillation on the risk of death: the Framingham Heart Study. Circulation.

[4] Lloyd-Jones D, Adams RJ, Brown TM, Carnethon M, Dai S, De Simone G (2010). American Heart Association Statistics Committee and Stroke Statistics Subcommittee-heart disease and stroke statistics-2010 update: a report from the American Heart Association. Circulation.

[5] Staerk L, Sherer JA, Ko D, Benjamin EJ, Helm RH (2017). Atrial Fibrillation: Epidemiology, Pathophysiology, and Clinical Outcomes. Circ Res.

[6] Miyasaka Y, Barnes ME, Gersh BJ, Cha SS, Bailey KR, Abhayaratna WP (2006). Secular trends in incidence of atrial fibrillation in Olmsted County, Minnesota, 1980 to 2000, and implications on the projections for future prevalence. Circulation.

[7] Heeringa J, van der Kuip DA, Hofman A, Kors JA, van Herpen G, Stricker BHC (2006). Prevalence, incidence and lifetime risk of atrial fibrillation: the Rotterdam study. European heart journal.

[8] Stewart S, Hart CL, Hole DJ, McMurray JJ (2001). Population prevalence, incidence, and predictors of atrial fibrillation in the Renfrew/Paisley study. Heart.

[9] Kannel WB, Abbott RD, Savage DD, McNamara PM (1982). Epidemiologic features of chronic atrial fibrillation: the Framingham study. N Engl J Med.

[10] Stambler BS, Ngunga LM (2015). Atrial fibrillation in Sub-Saharan Africa: epidemiology, unmet needs, and treatment options. Int J Gen Med.

[11] Ntep-Gweth M, Zimmermann M, Meiltz A, Kingue S, Ndobo P, Urban P (2010). Atrial fibrillation in Africa: clinical characteristics, prognosis, and adherence to guidelines in Cameroon. Europace.

[12] Chiang CE, Naditch-Brûlé L, Murin J, Goesthals M, Inoue H, Jose Silva-Cardosa JO (2012). Distribution and risk profile of paroxysmal, persistent, and permanent atrial fibrillation in routine clinical practice: insight from the real-life global survey evaluating patients with atrial fibrillation international registry. Circ Arrhythm Electrophysiol.

[13] Nieuwlaat R, Capucci A, Camm AJ, Olsson SB, Andresen D, Davies DW (2005). European Heart Survey Investigators, atrial fibrillation management: a prospective survey in ESC member countries: the Euro Heart Survey on atrial fibrillation. Eur Heart J.

[14] Zoni-Berisso M, Lercari F, Carazza T, Domenicucci S (2014). Epidemiology of atrial fibrillation: European perspective. Clin Epidemiol.

[15] Coulibaly S, Diall IB, Menta I, Diakité M, Ba HO, Sidibé S (2013). Atrial fibrillation in Cardiology Service of Point G Training Hospital: clinical, etiologic factors and natural evolution. Cardiologie tropical.

[16] Oldgren J, Healey JS, Ezekowitz M, Commerford P, Avezum A, Pais P (2014). RE-LY Atrial Fibrillation Registry Investigators. Variations in cause and management of atrial fibrillation in a prospective registry of 15 400 emergency department patients in 46 countries: the RE-LY Atrial Fibrillation Registry. Circulation.

[17] Shavadia J, Yonga G, Mwanzi S, Jinah A, Moriasi A, Otieno H (2013). Clinical characteristics and outcomes of atrial fibrillation and flutter at the Aga Khan University Hospital, Nairobi. Cardiovasc J Afr.

[18] Al-Khatib SM, Allen LNM, Chatterjee R, Crowley MJ, Dupre ME, Kong DF (2014). Rate-and rhythm-control therapies in patients with atrial fibrillation: a systematic review. Ann Intern Med.

[19] Kirchhof P, Benussi S, Kotecha D, Ahlsson A, Atar D, Casadei B (2016). Guidelines for the management of atrial fibrillation developed in collaboration with EACTS. European Heart Journal.

[20] Beyer-Westendorf J, Förster K, Pannach S, Ebertz F, Gelbricht V, Thieme C (2014). Rates, management, and outcome of rivaroxaban bleeding in daily care: results from the Dresden NOAC registry. Blood.

[21] Patel MR, Mahaffey KW, Garg J, Pan G, singer DE, Hacke W (2011). ROCKET AF Investigators Rivaroxaban versus warfarin in nonvalvular atrial fibrillation. N Engl J Med.

